# Not proportional after all: Investigating speed perception with the beep-speed illusion

**DOI:** 10.3758/s13414-025-03211-6

**Published:** 2026-01-13

**Authors:** Simon Merz, Joanna Sommerfeld, Christian Frings, Hauke S. Meyerhoff

**Affiliations:** 1 Department of Psychology, Cognitive Psychology & Institute for Cognitive and Affective Neuroscience, University of Trier, Universitätsring 15, 54286 Trier, Germany; 2 Institute for Cognitive and Affective Neuroscience, Trier, Germany; 3 Department of Psychology, Faculty of Human Sciences, University of Cologne, Cologne, Germany; 4 University of Erfurt, Erfurt, Germany

**Keywords:** Beep-speed illusion, Motion perception, Speed perception, Crossmodal, Audition, Vision

## Abstract

Recently, a new audiovisual illusion, the *beep-speed illusion*, was discovered in which a visually presented, linearly moving object is perceived to be faster if directional changes occur simultaneously with a short auditory burst compared to a purely visually presented moving object of the same speed. The present study uses this new illusion to test the representation of motion speed in human perception. Across two experiments (each N = 30), the beep-speed illusion was observed with circular motion of the objects and across different speeds of the audiovisual object. Interestingly, the size of the illusion, as well as the precision of the speed estimation as measured by the just notable difference, was not proportional in size to the audio-visual object speed across the different speeds. These results contradict predictions of proportionality in speed estimation derived from classical Weber law, and are discussed in light of recent theoretical developments in the field of motion/speed perception.

## Introduction

Our everyday experiences are multisensory in nature, for example we do not just see the ball being kicked by the footballer, but also hear the impact of the foot on the ball. Over the last decades, numerous studies have been conducted on the crossmodal influence on perception, offering new ways to explore human representation of the outside world (e.g., Chen & Spence, 2017; Ernst & Bülthoff, 2004; Koelewijn et al., 2010; Rahnev & Denison, 2018). Recently, a new illusion, the *beep-speed illusion*, was introduced, in which a moving object was perceived to be moving faster if unpredictable directional changes were accompanied by a brief auditory tone (Meyerhoff et al., 2022; see also Cañal-Bruland et al., 2022). The auditory tone seems to direct selective attention toward the coinciding direction-changing stimulus since comparable results were observed with a visual flash (Meyerhoff et al., 2022), and a simple response bias favoring the cued stimulus was ruled out as an alternative explanation (Meyerhoff et al., 2023). Intriguingly, the beep-speed illusion offers a new possibility to explore human speed perception for dynamic objects, a longstanding topic of discussion in the scientific literature (Park & Tadin, 2018; Stocker & Simoncelli, 2006; Zhang & Stocker, 2022).

The question regarding the representation of speed on motion perception in general has recently been refocused in the literature. In the motion localization literature, in which participants are tasked to indicate either perceived first (the Fröhlich effect & Onset-Repulsion effect: Fröhlich, 1923; Thornton, 2002) or final (the Representational Momentum & Offset repulsion effect: Freyd & Finke, 1984; Hubbard, 2005) stimulus location, new evidence indicates a curvilinear influence of stimulus speed on localization across a diverse stimulus set (across different motion trajectories, dependent variables and sensory modalities; Merz et al., 2019; Merz et al., 2022; see, Merz et al., 2025, for an extension to motion in depth; see Schroeger et al., (2022), for an extension to interception). This evidence shows resemblance with findings from the Thompson effect (Thompson, 1982), investigating the influences of contrast on motion speed, in which a perceived speed increase is only observed for slower speeds, but a reversal is observed for faster speeds (Thompson et al., 2006). This notion challenges classical findings of proportional constant relations of stimulus speed on motion perception. In fact, speed discrimination is thought to follow Weber fraction rules to be proportionally constant across a wide range of stimulus speeds (Bruyn & Orban, 1988; McKee & Nakayama, 1984), and neurophysiological evidence supports the possibility of such a proportional motion coding (Nover et al., 2005). Transforming the idea of proportionality to the beep speed illusion, it would be expected that the perceived speed increase induced by the beep-speed illusion is constant across different speeds. Even more, the discriminability of a stimulus being identical or different in speed, as measured by the just notable difference, should be proportional – that is, a constant ratio of needed speed increase and actual baseline speed across different speeds ([ΔSpeed/Baseline Speed] = K).

In order to allow for a substantial manipulation of stimulus speed, the experimental set-up and stimulus configurations need to be adapted compared to the original stimulus configurations (Meyerhoff et al., 2022, 2023). That is, previously, linearly moving stimuli were used, with unpredictable directional changes regarding the future stimulus motion direction. To circumvent the spatial limitations of the size of the screen for faster moving stimuli, a circular motion path was chosen. However, this change also introduced a new, potentially important change compared to the previous studies: The future direction of the stimulus was completely determined by its actual stimulus configuration, that is, a counterclockwise motion direction would change into a clockwise motion direction, and vice versa. Please note that this only corresponds to the predictability of the future spatial direction of the stimulus, but not the timing of when the stimulus will change its direction. Nevertheless, it is uncertain whether under these new stimulus configurations the beep-speed illusion can still reliably be observed, which is why, in Experiment 1, we conceptually replicated the experimental set-up with these new stimulus configurations, while for Experiment 2, we systematically manipulated the speed of the audiovisual stimulus to test the proportionality prediction of the Weber law.

### The present study

**Fig. 1:**
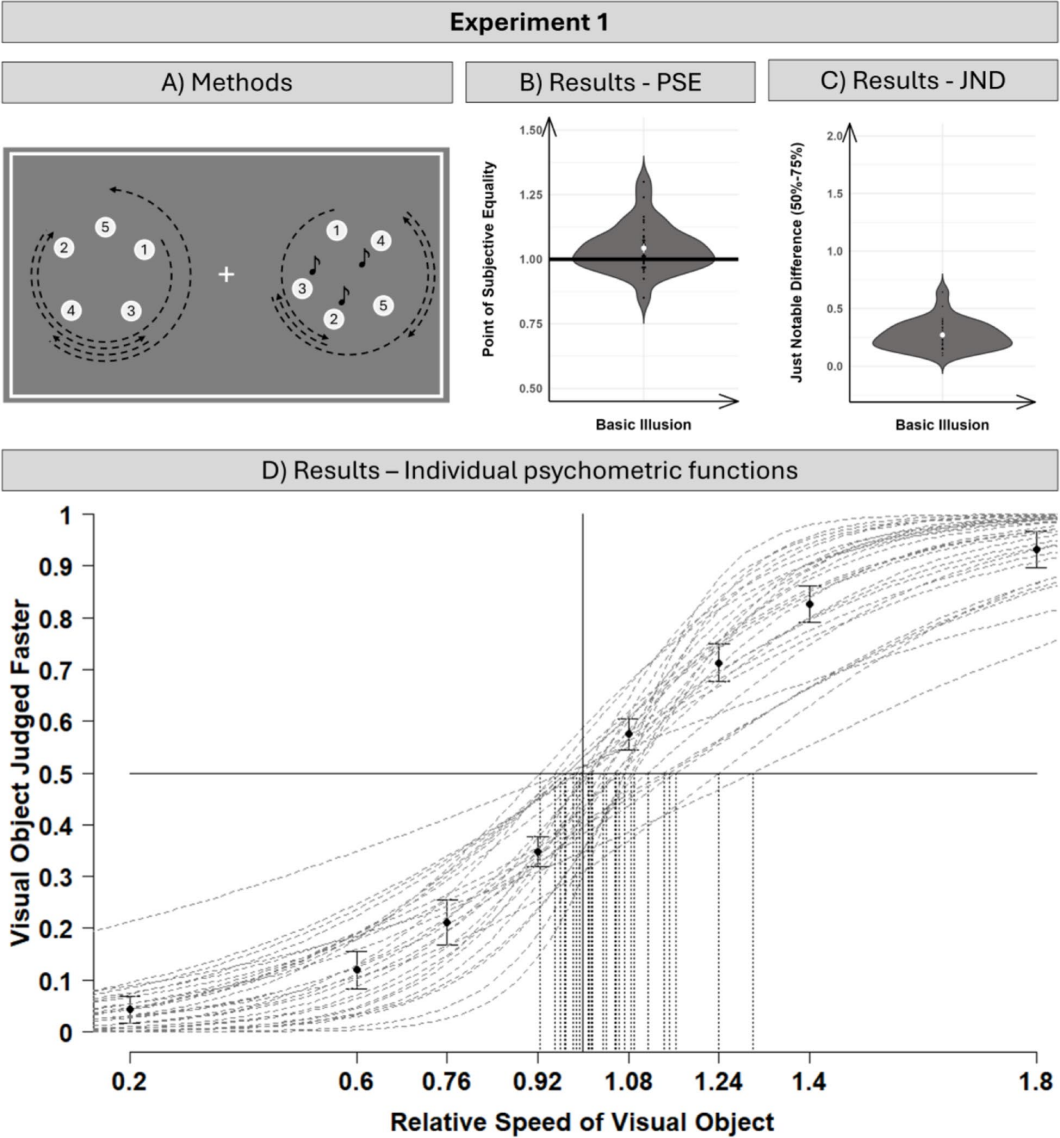
Visualization of the methods and results for Experiment 1. (**A**) Schematic display of the motion trajectory of the audiovisual target (on the right) and purely visual comparison stimulus (on the right). Five positions during an exemplary motion trajectory are visualized: starting location (1), first (2), second (3), and third (4) direction reversal, and ending position (5). For the audiovisual target, a non-spatial auditory tone (visualized with a note for illustration purposes – this note was not presented to the participants) was presented simultaneously to the direction reversal (positions 2–4). (**B/C**) Results of the calculated PSE (point of subjective equality) and JND (just noticeable difference) for each participant (black dots; white dot indicates average PSE/JND across participants). (**D**) Visualization of individual psychometric functions for each participant (dotted lines), with average judgments (dots; 95% CI following Morey, 2008) for each of the eight comparisons speeds used to calculate the psychometric functions. Dashed vertical lines indicate the PSE for each individually fitted psychometric function

In order to investigate human speed perception in the present study, participants were presented with two moving stimuli simultaneously: an audiovisual stimulus, for which each direction change was accompanied by an auditory beep sound, and a purely visual comparison stimulus without any additional sound (for a visualization, see Fig. 1A). The two stimuli were presented side-by-side simultaneously for 3 s and with three direction reversals for each trial. Participants were tasked with indicating whether the left or the right stimulus was perceived to move faster. An online-based data collection format was chosen as previous research has shown that speed perception and speed localization in general (Merz, 2022; Merz et al., 2022), and the beep-speed illusion in particular (Meyerhoff et al., 2023), can reliably be observed in online-base studies.

In order to test whether the changes of the stimulus set-up, especially the circular motion trajectory as well as the directional predictability of the future motion direction, had any influence on the occurrence of the beep-speed illusion, a conceptual replication was conducted in Experiment 1. Hereby, the speed of the audio-visual target stimulus was fixed at 0.25 rotations per second (rps), as this was approximated to be matching the stimulus speed used in previous studies (please note that the online nature of the experiments prevented an exact relation of the present experiment to the existing experiments). The speed of the visual comparison stimulus was systematically manipulated in eight steps to be faster/slower (ranging from 20% to 180% of the audiovisual object) in order to calculate the point of subjective equality (PSE) and just notable difference (JND). Please note for the interpretation that the extracted values for PSE and JND are relative scores (proportional to the audiovisual target speed), not absolute scores (e.g., rotations per seconds). This was done to facilitate comparability across different speeds (especially for Experiment 2 which used three different speeds for the audiovisual object), but also to test the proportionality prediction of Weber law. That is, following Weber law, it would be predicted that relative PSE/JND are identical across the different speeds.

## Experiment 1

### Method

#### Participants

The beep-speed illusion typically elicits medium to large effect sizes (d > 0.5; e.g., Meyerhoff et al., 2022), therefore a minimum sample size of at least 27 participants was targeted (α <.05; 1˗β >.80; power analyses were run with G-Power 3.1.9.2, option “means: difference from constant”; Faul et al., 2009). As the experiment was conducted online, and to account for possible drop-outs, 30 participants were tested, one of whom was excluded from the final sample (see details in the *Design, data preparation, and analysis* section*)*. The final sample (21 female, nine male, one left-handed; 19–29 years old, mean age: 22.31 years) consisted of 29 students from the University of Trier who participated for partial course credit. All the participants gave active informed consent prior to participation. The experimental procedures were designed in accordance with the regulations formulated in the Declaration of Helsinki; formal ethics approval was not required given the non-invasive and non-harmful experimental design.

#### Apparatus and stimuli

The experiment was conducted online and programmed with PsychoPy and its built-in online translation PsychoJS (Peirce et al., 2019). Data were collected via pavlovia.org. The participants were asked to use a computer or laptop of their choice; no tablets, touchscreens, or smartphones were allowed. If an operating system for a mobile device was detected, the experiment was discontinued.

Given the online character of the experiment, variance relating to the screen size was expected. Therefore, we coded all stimulus sizes relative to the height of the window using the corresponding PsychoPy3 unit "height" (i.e., a value of 1 corresponded to the full height of the window), and the experiment was run in full-screen mode. During trials, a visible white border (0.9 × 0.6 height) was presented, within which the two stimuli moved. The stimuli were two white dots (0.0125 height) in front of a gray background. The two circular trajectories of the stimuli were not visible, centered around a location 0.2 height to the left or right of the center of the screen, with the radius of the circular path fixed to 0.1 height. The auditory beep sound was a 440-Hz sine wave stimulus, presented for 68 ms. The speed of the audiovisual object was fixed at 0.25 rotations per second (rps), and the speed of the standard stimulus was one of eight speeds relating to the audiovisual object: 0.2, 0.6, 0.76, 0.92, 1.08, 1.24, 1.4, and 1.8. This corresponds to the following final speeds of the visual object: 0.05, 0.15, 0.19, 0.23, 0.27, 0.31, 0.35, and 0.45 rps).

#### Procedure

The trial procedure is visualized in Fig. 1. After a 600-ms starting interval, both stimuli were presented for 3 s, and within each interval of 1,000 ms, both objects changed their direction of motion once with the restriction that this change did not occur during the first or last 100 ms. Furthermore, it was ensured that the direction change of the audiovisual and visual stimulus was at least 200 ms apart so that the auditory beep sound was only simultaneous with the audiovisual stimulus, never with the visual stimulus. For each participant, the side of the objects (audiovisual left vs. right), the starting direction of the audiovisual object (clockwise vs. counterclockwise), and the relation between the starting direction of the audiovisual and visual object (same vs. different) were balanced. After the disappearance of the two objects, a response display was shown, and participants were asked to indicate whether the left or right object moved faster by pressing either the X (left faster) or M (right faster) key on the keyboard. Before the experimental trials started, the task was explained for the participants, and participants were given the chance to adapt the beeping sound to a comfortable level. In case of a multiple-loudspeaker set-up for the participants, the sound was presented across all speakers, and was therefore non-spatially informative. Subsequently, participants worked through eight practice trials, randomly selected from all possible experimental trials, and for the experiment, participants worked through 320 experimental trials (40 repetitions of each audiovisual and visual object velocity combination).

#### Design, data-preparation, and analysis

The participants were tested in a one-factorial design with the factor relative visual object speed (0.2, 0.6, 0.76, 0.92, 1.08, 1.24, 1.4, and 1.8). Participants were asked to indicate whether the left or right stimulus was faster. Therefore, in a first step, these binary responses were recoded into a binary value indicating whether the participants indicated the audio-visual object as moving faster than the visual object (1) or not (0). In a next step, for each participant individually, we checked for each participant in each condition whether the responses varied with the objective differences in the speed of the visual object (otherwise this would indicate random response behavior). To do so, we fitted the binary variable indicating whether the participants reported the audio-visual object as moving faster than the visual object using generalized linear models with logit linking functions. We fitted two models for each participant. The first model only included an intercept representing the general tendency to mark the audio-visual object as moving faster. The second model included the intercept and a slope for the objective speed of the visual object. For all participants and in each condition, the model with the slope explained the data significantly better (i.e., the increase in explained variance was worth adding the additional parameter as supported by a significant (*p* <.05) chi-square test when both models are compared). Therefore, no participant was excluded. From the models with the slope parameter, we then calculated the PSE (i.e., the speed value of the visual object at which both objects appear to move at the same speed) as well as the JND (i.e., the difference between the speed conditions in which – following the fitted psychometric function – 50% and 75% discrimination accuracy occurred) for each participant. To calculate the PSE for each participant individually, the negative intercept was divided by the slope parameter, while for the 50–75% JND, the difference between the relative visual speed scores corresponding to the 50% and 75% visual object judged faster response (extracted from the individual psychometric functions) was divided by the slope parameter. For the calculated PSE and JNDs, we checked whether any individual PSE/JND deviated by more than 3 SD from the average PSE, which did occur for one participant (individual JND of 1.84; group mean: 0.32, standard deviation: 0.31); therefore this participant was excluded.^1^ The extracted PSE was tested against 1, as this corresponds to the speed of the audiovisual object, and 95% confidence interval (CI) of effect sizes was reported using JASP (version 0.19.3). All experimental files, code, and data can be found on the project’s Open Science Framework (OSF) page.

### Results and discussion

As Fig. 1 shows, we observed the beep-speed illusion with our new set-up, with a average JND of about 0.27, and a shift of the PSE toward an increased speed of the visual object (*M* = 1.05; t-test comparison against 1 – no perceptual increase), *t*(28) = 3.08, *p* = .005, *d* = 0.57; 95% CI of effect size [0.18, 0.96]. In other words, the visual stimulus needed to be approximately 27% faster than the audiovisual stimulus to be likely perceived to be faster, as determined by the JND, and the visual object had to move approximately 5% faster than the audiovisual object to appear to be equally fast. This replicates the existence of the beep-speed illusion, as previously observed with linear motion, in this case with circular motion. Interestingly, the size of the illusion is somewhat comparable to the size of the illusion observed in previous studies (approximately 4–5%). Furthermore, this indicates that the uncertainty about motion trajectory following the change of direction is not an important precondition for the illusion to occur. Most importantly, the results of Experiment 1 allowed us to conduct Experiment 2, in which the audiovisual object was presented with differing speeds.

## Experiment 2

### Method

#### Participants

Considerations for sample size were identical to Experiment 1; as no previous study has estimated the size of any effect of speed on the beep-speed illusion, sample size considerations focused on the observation of the pure beep-speed illusion. Please also note that in Experiment 1, a medium effect size (*d* of 0.47) was also observed, in line with our a priori sample size considerations. The final sample (16 female, 13 male; three left-handed; 19–32 years old, mean age: 22.59 years) consisted of a total of 29 participants from the University of Trier who participated for partial course credit (see *data preparation*). All the participants gave active informed consent prior to participation.

#### Apparatus and stimuli, procedure, design, data preparation, and analysis

Experiment 2 was identical to Experiment 1 except for the following changes. Besides the velocity of 0.25 rps of the audiovisual object, the audiovisual object was presented in two further velocities: one slower velocity of $0.041\overline{6}$ rps (1/24 rps) and one faster velocity of 1.5 rps. The velocities of the corresponding visual object were identical to Experiment 1 (0.2, 0.6, 0.76, 0.92, 1.08, 1.24, 1.4, and 1.8), resulting in a range of absolute speeds from $0.008\overline{3}$ to 0.075 rps for the slower audiovisual object ($0.041\overline{6}$ rps), and from 0.3 to 2.7 rps for the faster audiovisual object (1.5 rps). Overall, participants worked through 768 randomly presented experimental trials (32 repetitions of each audiovisual and visual object velocity combination for each of the three audiovisual object velocities). Due to the same data preparation criteria as in Experiment 1, the calculated PSE of one participant was very high and deviated more than 3 standard deviations from the mean in two of the three speed conditions, therefore this participant was excluded from data analysis.^2^

### Results and discussion

**Fig. 2:**
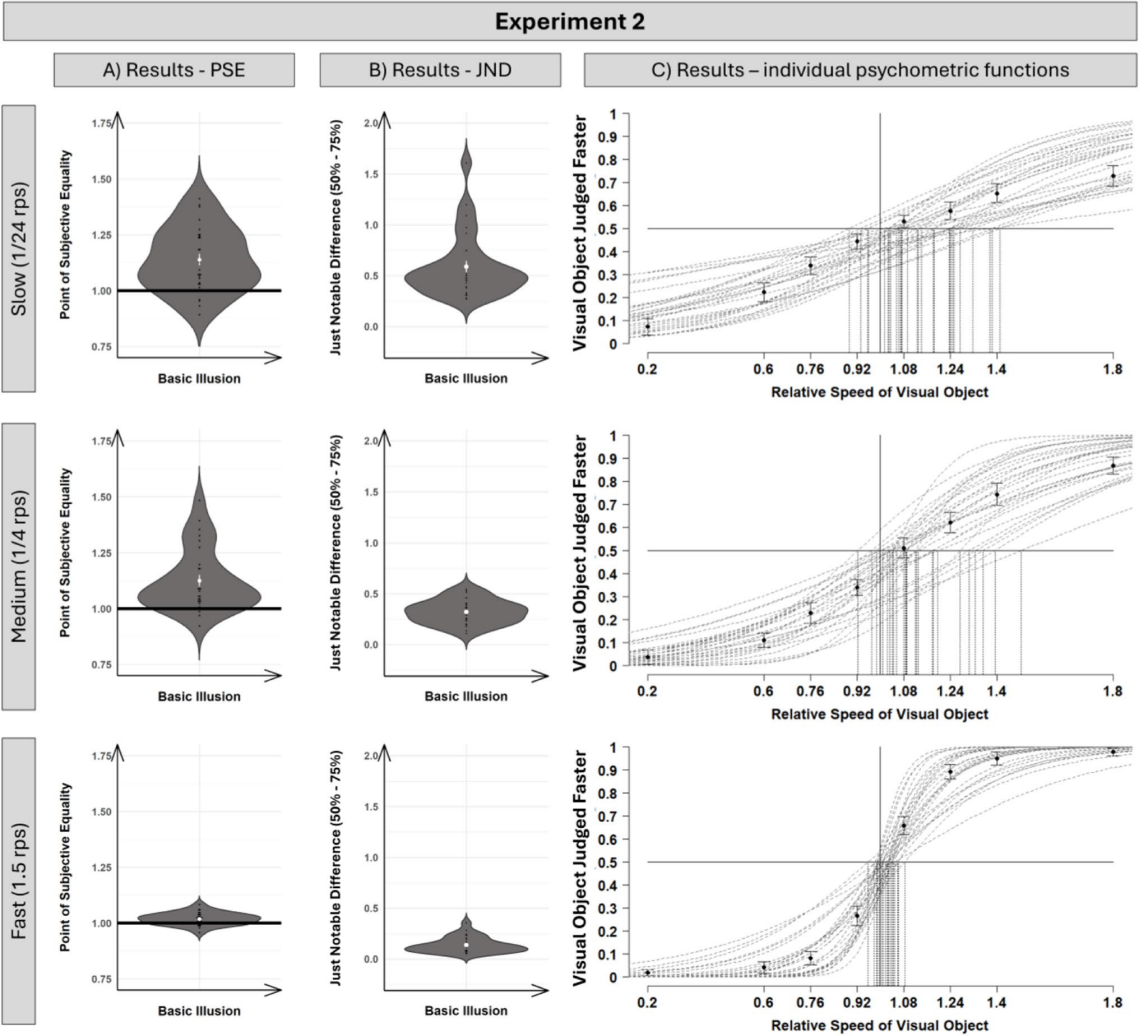
Visualization of the results of Experiment 2 on the slow (upper), medium (middle), and fast (lower) speed conditions. (**A/B**) Results of the calculated PSE (point of subjective equality) and JND (just noticeable difference) for each participant (black dots; white dot indicates average PSE across participants). (**C**) Visualization of individual psychometric functions for each participant (dotted lines), with average judgments (dots; standard errors following Morey, 2008) for each of the eight comparison speeds used to calculate the psychometric functions. Dashed vertical lines indicate the PSE for each individually fitted psychometric function

As Fig. 2 indicates, we observed the beep-speed illusion for the slow (*M* = 1.14), *t*(28) = 5.34, *p* < .001, *d* = 1.00; 95% CI [0.54, 1.43], medium (*M* = 1.13), *t*(28) = 4.85, *p* < .001, *d* = 0.90; 95% CI [0.46, 1.33], and fast (*M* = 1.02), *t*(28) = 3.73, *p* < .001, *d* = 0.69; 95% CI [0.28, 1.09] audio-visual object speeds. However, the relative size of the shifts was not comparable across the three different audiovisual speeds as supported by a significant main effect of audio-visual target speed in a one-factorial repeated-measure ANOVA with participants’ PSE as the dependent variable. A significant main effect of audiovisual object speed was observed, *F*(2, 56) = 15.89, *p* < .001, ɳ_p_^2^ = .36, and Helmert contrasts reveal a significant difference between the fast and the average of the medium and slow conditions, *t*(28) = 4.91, *p* < .001, while no difference between the medium and slow conditions was observed, *t*(28) = 0.70, *p* = .487. Therefore, the size of the PSE was not proportional to the speed of the audiovisual object stimulus.

Similarly, for the JND values, a significant main effect of audio-visual target speed was observed, *F*(1.15, 32.10) = 57.80, *p* < .001, ɳ_p_^2^ = .67, with best discrimination performance for the fastest speed condition (0.14), and the worst for the slowest speed condition (0.59), both deviating significantly from the medium speed condition (0.32), as revealed by two simple contrasts (fast vs. medium: *t*(28) = 11.45, *p* < .001, *d* = 0.94; medium vs. slow: *t*(28) = 5.49, *p* < .001, *d* = 1.37). That is, only a 14% speed increase was needed for the fast speed condition for a difference to be perceived, whereas for the slow speed condition, a 59% speed increase was necessary. This, comparable to the PSE results, indicates that the speed discrimination performance was not proportionally constant from absolute speed, contradicting Weber’s law formulation of proportionality for speed discrimination.

## General discussion

The present study was designed to investigate speed perception with the help of the newly discovered beep-speed illusion. As this necessitated several deviations from the classical experimental task (Meyerhoff et al., 2022, 2023) such as circular instead of linear motion, and predictability of the future motion direction following the direction change, Experiment 1 was conducted to replicate the basic beep-speed illusion under these new stimulus configurations. Hereby, a reliable beep-speed illusion was observed, indicating the robustness of the illusion beyond a specific stimulus configuration, similar to other perceptual motion biases (e.g., Representational Momentum: Hubbard, 2018b; Fröhlich effect: Müsseler & Kerzel, 2018; Flash lag/lead: Hubbard, 2018a). This is in line with formulations arguing for the relevance of (selective) visual attention for audio-visual integration to occur (Meyerhoff et al., 2022; see also Jensen et al., 2019; Koelewijn et al., 2010; Merz et al., 2021; Spence & Frings, 2020; Talsma et al., 2010).

The main experiment was designed to analyze the size of the beep-speed illusion across different speeds to test the idea of proportionality of speed perception across different speed ranges. Interestingly, the proportional size of the illusion was only comparable for the medium and slow condition (approximately 13–14%), while for the fast condition, the size of the illusion was considerably reduced (approximately 2%). Similarly, and even more surprising, the speed discrimination performance was not proportional to the absolute speed of the audiovisual stimulus. That is, a relative moderate (approximately 14%) speed increase was enough to be detected for the fast stimulus condition, while the speed increase needed to be more than threefold for the slow speed condition to be detectable (59%). These findings are not in line with the idea of proportional constant speed discriminations following Weber fraction rules (Bruyn & Orban, 1988; Nover et al., 2005).

Interestingly, recent formulations about speed perception (Merz et al., 2022), arguing for the importance of speed expectations to inform speed perception, might offer an explanation for the current findings. That is, they argue that deviations between expected and actual speed result in deviations of perceived from actual speed, as noisy sensory input is combined with expectations to reduce uncertainty at any given moment (Merz et al., 2022; for more on the general argument of this “bayesian brain idea,” see Clark, 2013; Friston, 2005; Knill & Pouget, 2004). Crucially, as the relation between actual and expected target speed is different for the different speed conditions, non-linear and non-proportional associations between the size of the illusion, speed discrimination, and the different target speeds should be observed, as our results indicate (for a more extended discussion, see Merz et al., 2025; Merz et al., 2022).

One further detail of the present results should also be considered in this discussion. That is, the experimental conditions of Experiment 1 were exactly identical to the medium speed condition of Experiment 2. In other words, the same stimulus configurations/comparison speeds were used in both conditions, yet, remarkably, the size of the observed beep-speed illusion differed strongly (5.0% in Experiment 1, 12.5% in the medium speed condition in Experiment 2), even though speed discriminability was not affected. An exploratory, across-experiment t-test comparison revealed the difference between the shift of the PSE to be statistically significant (difference: 7.5%, *t*(56) = 2.47, *p* =.016, *d* = 0.65, CI [0.12, 1.17]), while the JND did not significantly differ (difference: 0.05, *t*(56) = 1.62, *p* =.111, *d* = 0.43, CI [0.10, 0.94]). The question arises why these identical trial types lead to such different perceptions regarding the size of the illusion. Theoretical formulations arguing for the relevance of speed expectations, as outlined in the previous paragraph, might offer an explanation. That is, as in Experiment 2, the other two speed conditions (the fast and slow condition) were not presented in different blocks, but within the same block, and the trials were selected at random from all possible trial types, this might have led to a different speed expectation in Experiment 2 compared to Experiment 1. In fact, there are several reports of different perceptual performance for identical trials due to extensive learning of a specific speed (Sotiropoulos et al., 2014) or manipulations of the speed profile within one experimental context (Merz et al., 2023, 2024). This idea would be further in line with recent findings that our perceptual system is able to extract a high detail of variability within an experimental context (for discussion and overview, see Brady & Alvarez, 2011; Chetverikov & Kristjánsson, 2024; Whitney & Yamanashi Leib, 2018), allowing for the context-dependent formation of speed expectations.

To conclude, the present study extends the beep-speed illusion to new experimental conditions, highlighting the generalizability of the illusion across different settings and contexts. However, more importantly, our results indicate a non-proportional change in the size of the beep-speed illusion across different speeds, against the prediction of Weber law rules, and we discuss the strong potential of Bayesian brain formulation, arguing for the importance of speed expectations informing perceptual experience, to explain the present results.

## Data Availability

Data and materials are available via the Open Science Framework (OSF) repository at: https://osf.io/vjrnm/?view_only=2aa7990f0fd94ed895549e3360598c8c

## Footnotes

1 Please note that the exclusion of this participant did not significantly change the interpretation of the observed results reported in the main text.

2 Please note that the exclusion of this participant did not significantly change the interpretation of the observed results reported in the main text.

## Code availability

Code is available via the Open Science Framework (OSF) repository at: https://osf.io/vjrnm/?view_only=2aa7990f0fd94ed895549e3360598c8c
